# Atypical Pediatric Epilepsy: A Narrative Review of Classification, Diagnosis, and Emerging Therapeutics

**DOI:** 10.7759/cureus.117344

**Published:** 2026-10-02

**Authors:** Migdalia Iglesias, Kevin Sherin

**Affiliations:** 1 Family Medicine and Rural Health, Florida State University College of Medicine, Sarasota, USA; 2 Family Medicine and Rural Health, Florida State University College of Medicine, Orlando, USA

**Keywords:** atypical epilepsy, autoimmune epilepsy, developmental and epileptic encephalopathy, dravet syndrome, fires, genetic epilepsy, ilae classification, infantile epileptic spasms syndrome, lennox-gastaut syndrome, pediatric epilepsy

## Abstract

Atypical pediatric epilepsies represent a heterogeneous group of disorders characterized by uncommon etiologies, variable clinical presentations, and frequent resistance to standard antiseizure medications. Recent advances in classification, along with developments in genomics, neuroimaging, and autoimmune neurology, have expanded the recognized spectrum of these conditions. However, a significant proportion of children remain without a definitive syndromic diagnosis, underscoring ongoing limitations in current frameworks.

This review provides a comprehensive overview of atypical epilepsy in children, including evolving classification systems, epidemiology, and etiological mechanisms spanning genetic, structural, metabolic, and immune-mediated causes. Key syndromes - including Lennox-Gastaut syndrome, Dravet syndrome, infantile epileptic spasms syndrome, and febrile infection-related epilepsy syndrome - are discussed with emphasis on clinical features, differential diagnosis, and prognosis. Diagnostic challenges such as phenotypic overlap, EEG limitations, and barriers to timely genetic testing are also examined.

Emerging therapeutic strategies highlight a paradigm shift from broad-spectrum seizure control toward precision medicine, including targeted pharmacologic agents, antisense oligonucleotides, gene therapies, and neuromodulation approaches. Improved recognition and classification of atypical epilepsies, coupled with early implementation of targeted therapies, are essential to optimize outcomes in this vulnerable population.

## Introduction and background

Epilepsy is the most prevalent chronic neurological disorder in children, with an incidence of 41 to 187 cases per 100,000 population. First-line management consists of antiseizure medications (ASMs); however, the incidence of drug-resistant childhood epilepsy is approximately 15%. Certain childhood epilepsy syndromes, such as developmental and epileptic encephalopathies (DEEs) - a group of conditions in which epileptic activity and/or the underlying disorder contribute to developmental impairment - have a natural tendency to be drug-resistant [1]. Drug-resistant epilepsy (DRE) forms a big part of the atypical childhood epilepsy syndromes. Nevertheless, the concept of “atypical” pediatric epilepsy is multifaceted and encompasses: (1) rare epilepsy syndromes with low population incidence; (2) atypical presentations of otherwise common syndromes; (3) epilepsies with unusual etiologies (autoimmune, metabolic); (4) unusual seizure types (reflex seizures, non-convulsive status); and (5) epilepsies that blur the boundaries between developmental encephalopathy and epileptic encephalopathy.

The 2022 International League Against Epilepsy (ILAE) Task Force provided, for the first time, formally accepted definitions for epilepsy syndromes across the pediatric age spectrum [2-5]. Simultaneously, advances in genomics, autoimmune neurology, and neuroimaging have expanded the recognized etiological spectrum. A Norwegian population-based cohort study found that a defined ILAE epilepsy syndrome could be identified in only 53% of children and youth with epilepsy, while a substantial proportion remained without a syndromic diagnosis [6]. These findings underscore both the utility and the limitations of current classification systems in capturing the full spectrum of pediatric epilepsy presentations.

The purpose of this review is to synthesize current evidence on atypical pediatric epilepsies, with an emphasis on evolving classification systems, etiological heterogeneity, diagnostic challenges, and emerging therapeutic strategies. For the purposes of this narrative review, the term “atypical pediatric epilepsy” is used as an umbrella concept rather than as a formal ILAE diagnostic category. Given the rapid expansion of knowledge in genomics, autoimmune neurology, and precision therapeutics, this review aims to integrate recent advances into a clinically meaningful framework for recognizing and managing atypical epilepsy presentations in children. This review is intended for clinicians involved in the care of pediatric patients with epilepsy, including pediatric neurologists, epileptologists, and pediatricians, as well as researchers interested in epilepsy classification and emerging therapies.

## Review

Methods

This study is a narrative review of the literature focusing on atypical pediatric epilepsies. A structured literature search of PubMed, Embase, and Google Scholar was performed for literature published between 2019 and 2026, with emphasis on recent advances in classification, diagnostics, and therapeutics. The initial literature search was completed in June 2026 and was subsequently updated through September 27, 2026, to capture recently published studies before manuscript revision.

Search terms included combinations of the following keywords: “atypical pediatric epilepsy”, “developmental and epileptic encephalopathies”, “epilepsy classification”, “genetic epilepsy”, “epilepsy diagnosis”, “drug-resistant epilepsy”, “precision medicine”, and “emerging therapeutics”. Search terms were combined using the Boolean operators AND and OR and adapted to the syntax of each database. The principal PubMed search strategy combined epilepsy-related terms (“epilepsy” OR “epileptic encephalopathy” OR “developmental and epileptic encephalopathy”) with pediatric terms (“pediatric” OR “child” OR “infant” OR “adolescent”) and topic-specific terms (“atypical” OR “classification” OR “syndrome” OR “genetic” OR “diagnosis” OR “drug-resistant” OR “precision medicine” OR “therapy” OR “autoimmune” OR “immune-mediated”), with publication dates restricted to January 1, 2019 through September 27, 2026. The same conceptual search blocks were adapted for Embase. Google Scholar was used as a supplementary broad search using combinations of the same terms and the 2019-2026 date range.

Articles were selected based on relevance to the scope of the review, with priority given to systematic reviews, meta-analyses, clinical trials, and cohort studies. Studies were considered eligible if they addressed pediatric epilepsy and provided information relevant to at least one of the principal topics of this review: epilepsy classification, epidemiology, etiology or genetics, diagnostic challenges, syndrome-specific clinical features, or emerging therapeutic approaches. ILAE position statements and other authoritative guidance documents were also included when relevant to classification or clinical terminology. Studies were excluded if they focused exclusively on adult populations without separately reported pediatric data, did not address epilepsy, or were not relevant to the predefined scope of the review. Highly relevant reports of emerging precision therapies were retained when they provided clinically important pediatric data despite small sample sizes. Because this was a narrative rather than systematic review, study selection was guided by relevance to the review objectives rather than by a formal systematic-review protocol.

Relevant information was extracted narratively and organized according to the major domains of the review, including classification, epidemiology, etiology and genetics, syndrome-specific characteristics, diagnostic challenges, and therapeutic advances. Numerical estimates - including incidence and prevalence, diagnostic yields, treatment-response rates, and other effect estimates - were narratively summarized from the individual source publications rather than recalculated or pooled across studies.

2022 ILAE classification - defining the atypical in children

Neonatal and Infantile Syndromes

The ILAE Task Force on Nosology and Definitions proposed a classification of neonate and infant epilepsy syndromes with seizure onset up to two years old [2]. Syndromes were separated into self-limited syndromes, where spontaneous remission is likely, and DEEs, where developmental impairment is both the underlying etiology and the epileptic encephalopathy [2]. Additionally, an emerging class of etiology-specific syndromes, where there is a specific etiology associated with a clearly defined clinical phenotype, was presented [2]. This classification reflects a shift toward integrating etiology with electroclinical features, which is particularly relevant in atypical and genetically mediated epilepsies.

Self-limited neonatal epilepsy syndromes are due to pathogenic variants, most commonly KCNQ2 and less commonly KCNQ3 or SCN2A [7]. Self-limited neonatal epilepsy syndromes include self-limited (familial) neonatal epilepsy (SeLNE), self-limited familial neonatal-infantile epilepsy (SeLFNIE), self-limited (familial) infantile epilepsy (SeLIE), genetic epilepsy with febrile seizures plus (GEFS+) spectrum, and myoclonic epilepsy in infancy (MEI) [2].

Neonatal and infantile DEEs include early-infantile DEE (EIDEE), epilepsy of infancy with migrating focal seizures (EIMFS), infantile epileptic spasms syndrome (IESS), and Dravet syndrome (DS), previously known as severe myoclonic epilepsy of infancy [2]. Three additional DEEs have been described, consisting of KCNQ2-associated DEE, pyridoxine-dependent and pyridoxal phosphate deficiency-associated DEE, and CDKL5-associated DEE [7]. These three DEEs are also classified as etiology-specific syndromes. Additional etiology-specific syndromes include PCDH19 clustering epilepsy, glucose transporter 1 deficiency syndrome (Glut1DS), Sturge-Weber syndrome, and gelastic seizures with hypothalamic hamartoma [2]. Collectively, these syndromes highlight the early onset, genetic heterogeneity, and often severe clinical course that characterize atypical epilepsies in this age group.

Childhood-Onset Syndromes

The ILAE Task Force on Nosology and Definitions proposed a classification of epilepsy syndromes of childhood-onset (age 2-12 years). Syndromes were divided into self-limited focal epilepsies (SeLFEs), genetic generalized epilepsies, and DEEs, which can consist of focal or generalized seizures or seizures that are both focal and generalized [3].

SeLFEs are further divided into Group 1, consisting of self-limited epilepsy with centrotemporal spikes (SeLECTS) and self-limited epilepsy with autonomic seizures (SeLEAS), and Group 2, composed of childhood occipital visual epilepsy (COVE) and photosensitive occipital lobe epilepsy (POLE) [3]. Genetic generalized epilepsies consist of childhood absence epilepsy (CAE), epilepsy with myoclonic absence (EMA), epilepsy with eyelid myoclonia (EEM), previously known as Jeavons syndrome, and epilepsy with myoclonic-atonic seizures (EMAtS), which is also classified as a DEE [3]. Childhood-onset DEEs further consist of Lennox-Gastaut syndrome (LGS), DEE with spike-and-wave activation in sleep (DEE-SWAS) and EE-SWAS, febrile infection-related epilepsy syndrome (FIRES), and hemiconvulsion-hemiplegia-epilepsy syndrome (HHE) [3]. This classification underscores the diagnostic complexity of pediatric epilepsy, where syndromes may evolve over time or share overlapping electroclinical features.

Epidemiology and etiology of rare and atypical pediatric epilepsies

Epidemiological studies demonstrate substantial heterogeneity in the incidence and prevalence of atypical pediatric epilepsies. Population-based data indicate that DEEs collectively represent a substantial proportion of childhood epilepsy, although individual rare epilepsy syndromes vary markedly in frequency [6,8,9]. Syndrome-specific studies further demonstrate differences in incidence, prevalence, and mortality among disorders such as DS, LGS, IESS, and other severe epilepsies of infancy [9-11]. Genetic epidemiological studies also highlight the contribution of single-gene disorders to childhood-onset epilepsy [12]. Key epidemiological findings from population-based and syndrome-specific studies are summarized in Table 1.

**Table 1:** Epidemiological Characteristics of Selected Atypical Pediatric Epilepsies Data were synthesized from population-based and systematic epidemiological studies of childhood epilepsy syndromes [6], rare epilepsies [8], DEEs [9], DS and LGS [10], severe epilepsies of infancy [11], and childhood-onset genetic epilepsies [12]. D/EE-SWAS: developmental and/or epileptic encephalopathy with spike-and-wave activation in sleep; DEE: developmental and epileptic encephalopathy; DS: Dravet syndrome; EIEE: early infantile epileptic encephalopathy; EIMFS: epilepsy of infancy with migrating focal seizures; EMAtS: epilepsy with myoclonic-atonic seizures; IESS: infantile epileptic spasms syndrome; LGS: Lennox-Gastaut syndrome; SUDEP: sudden unexpected death in epilepsy; TSC: tuberous sclerosis complex; WS: West syndrome

Population/Syndrome	Epidemiologic Findings	Key Findings/Significance
Childhood epilepsy syndromes	Cumulative incidence per 1,000 children: self-limited epilepsies, 2.25; idiopathic generalized epilepsies, 1.75; DEEs, 2.62	DEEs collectively represent a substantial proportion of childhood epilepsy
Rare epilepsies (New York City)	IESS: 1 in 2,920 live births; LGS: 1 in 9,690; TSC-associated seizures: 1 in 14,300	IESS, LGS, and TSC-associated seizures were among the most common rare epilepsies studied
Other rare syndromes (New York City)	D/EE-SWAS: 1 in 34,100; Sturge-Weber syndrome: 1 in 27,800-40,900; EIMFS: 1 in 22,200-247,000; hypothalamic hamartoma: 1 in 52,200-225,000; Rasmussen syndrome: 1 in 450,000-652,000	Demonstrates the marked variability in incidence among individual rare epilepsy syndromes
DEEs (New Zealand)	Cumulative incidence: 169 per 100,000 children; IESS: 58.2; EMAtS: 16.4; LGS: 13.2; DS: 5.1 per 100,000	One-third of children with DEE could not be diagnosed with a known epilepsy syndrome
DS	Incidence: 2.2-6.5 per 100,000; prevalence: 1.2-6.5 per 100,000; mortality: 15.84 per 1,000 person-years	SUDEP was the most frequently reported cause of death
LGS	Incidence: 14.5-28 per 100,000; prevalence: 5.8-60.8 per 100,000; mortality: 6.12 per 1,000 person-years	Incidence and prevalence estimates were generally higher for LGS than DS, while mortality was higher for DS
Severe epilepsies of infancy (Australia)	IESS/“WS-like” epilepsy: 32.7 per 100,000 live births/year; EIMFS: 4.5 per 100,000; EIEE: 3.6 per 100,000	Approximately three-quarters could be assigned an epilepsy syndrome or “variant syndrome” at presentation
Childhood-onset genetic epilepsies	Overall annual incidence of single-gene epilepsies: 47.2 per 100,000; PRRT2: 10.0; SCN1A: 8.26; KCNQ2: 5.89; SLC2A1: 4.13 per 100,000	PRRT2 was the most common single-gene epilepsy reported in the cohort

The etiologies of atypical pediatric epilepsies are heterogeneous and include genetic, structural, metabolic, infectious, and immune-mediated causes [1]. Structural etiologies may be genetic or acquired and include developmental malformations, tumors, and vascular insults. Genetic etiologies are predominantly monogenic but also include polygenic epilepsies, such as SeLECTS, GEFS+, and POLE, and non-Mendelian chromosomal conditions, including 1p36 deletion, 15q duplication, trisomy 21, and ring chromosome 20 syndrome [1]. Metabolic causes include inborn errors of metabolism and mitochondrial disorders [1]. Representative disorders and molecular associations are summarized in Table 2.

**Table 2:** Representative Etiologies and Molecular Associations in Atypical Pediatric Epilepsies FIRES: febrile infection-related epilepsy syndrome; NMDA: N-methyl-D-aspartate

Etiologic Category	Representative Disorders/Epilepsy Phenotypes	Representative Genes or Mechanisms
Genetic - monogenic	Self-limited neonatal/infantile epilepsies; developmental and epileptic encephalopathies (DEEs); familial focal epilepsies	KCNQ2, KCNQ3, SCN1A, SCN2A, SCN8A, PRRT2, PCDH19, STXBP1, KCNT1, CDKL5, MECP2, LGI1, DEPDC5
Genetic - structural	Lissencephaly; focal cortical dysplasia; tuberous sclerosis complex	ARX, DCX, PAFAH1B1, MTOR, PIK3CA, AKT3, DEPDC5, TSC1/TSC2
Metabolic	GLUT-1 deficiency; pyridoxine-dependent epilepsy; biotinidase deficiency	SLC2A1, ALDH7A1, BTD
Mitochondrial	Mitochondrial disorders associated with epilepsy	POLG, RRM2B, SLC25A22, PDHA1, MTND1
Immune-mediated/inflammatory	FIRES; anti-NMDA receptor encephalitis; Rasmussen syndrome	Cytokine-mediated, autoantibody-mediated, and T-cell-mediated mechanisms

Syndrome-specific profiles

Epilepsy With Myoclonic-Atonic Seizures (EMAtS)

EMAtS is a childhood-onset epilepsy syndrome characterized by high prognostic and genetic variability. The syndrome can also include absence and generalized tonic-clonic seizures, and developmental delay often occurs during seizure exacerbation. Guerrini et al. (2025) provided an updated review of EMAtS, addressing nosological overlaps with other seizure disorders such as LGS and DEE-SWAS [13]. Distinguishing EMAtS from other childhood-onset syndromes with generalized seizures can be challenging. A key differential diagnosis is late-infantile neuronal ceroid lipofuscinosis type 2 (CLN2), which presents with typical development or isolated speech delay but shows rapid motor and cognitive decline. Photosensitivity testing at very low frequencies (e.g., 1 Hz) is crucial for differential diagnosis, as a photoparoxysmal response at low frequencies is a hallmark of CLN2 but rare in EMAtS [13]. DEE-SWAS is very difficult to differentiate from EMAtS based on presentation alone, but EEG-electromyography shows no myoclonic-atonic seizures. LGS can be distinguished from EMAtS by the early occurrence of tonic seizures, absence of myoclonic status epilepticus, and EEG showing slow (2.5 Hz) generalized spike-wave complexes with paroxysmal fast activity during sleep [13].

Lennox-Gastaut Syndrome (LGS)

LGS is a severe DEE with a typical onset between three and five years of age. LGS has high etiological variability, and causes may be identified in around 75% of cases, which can be divided into structural, genetic, metabolic, or combined [14]. Multiple de novo single-nucleotide variants have been associated with LGS. Nevertheless, the genetic profiles between different phenotypes can greatly overlap, as in many cases LGS could develop from IESS. Consequently, there is no single causative gene; rather, LGS reflects convergence across multiple DEE-related genetic pathways [14]. Secondary LGS accounts for approximately 60% of cases and is associated with underlying brain pathology [15]. Common causes include tuberous sclerosis, CNS infections, frontal lobe injuries, and developmental malformations. About 25% of patients who develop LGS have a history of infantile spasms, often leading to a more severe disease course [15]. EEG demonstrates generalized slow spike-and-wave complexes of 2.5 Hz or less and generalized paroxysmal fast activity during sleep. Seizures are often treatment-resistant, necessitating multimodal therapy such as dietary therapy and surgical or neuromodulatory interventions [15].

Febrile Infection-Related Epilepsy Syndrome (FIRES)

FIRES is a subcategory of new-onset refractory status epilepticus (NORSE) characterized by the development of refractory seizures following a nonspecific febrile illness. Seizures typically begin within 24 hours to two weeks after the febrile illness, often progressing rapidly to super-refractory status epilepticus [16]. The etiology of most FIRES cases remains cryptogenic, posing a significant challenge to clinicians. When a cause is identified, it most commonly falls within the categories of autoimmune encephalitis or genetic disorders. However, the absence of identifiable neural autoantibodies in the majority of cases distinguishes FIRES from classical antibody-mediated autoimmune encephalitis [16]. Emerging evidence points toward autoinflammation as a unifying pathophysiological mechanism in cryptogenic cases. Long-term outcomes are poor, with high rates of cognitive impairment and functional disability [16]. Management requires aggressive treatment of refractory status epilepticus, and current evidence supports the use of immunomodulatory therapy in cryptogenic NORSE/FIRES; recent consensus recommendations also incorporate early ketogenic diet therapy (KDT) as part of the treatment approach [16].

D/EE With Spike-and-Wave Activation in Sleep (D/EE-SWAS)

D/EE-SWAS, formerly known as encephalopathy with status epilepticus during slow sleep (ESES/CSWS), is a rare but severe epilepsy syndrome characterized by sleep-potentiated epileptiform discharges, seizures, and progressive cognitive, behavioral, and motor regression [17]. Its etiology is heterogeneous, encompassing pathogenic genetic variants - most notably in GRIN2A - and structural thalamocortical lesions, though many cases remain cryptogenic. It is important to differentiate Landau-Kleffner syndrome (LKS) from ESES since both share the same EEG pattern [17]. LKS presents with isolated auditory verbal agnosia - rather than global regression - and fewer seizures. Early recognition is critical, as prompt treatment with corticosteroids, benzodiazepines, or other immunomodulatory therapies may improve cognitive outcomes [17].

Dravet Syndrome (DS)

DS is a severe DEE that can lead to various epilepsy syndromes, such as GEFS+ syndromes. It is mostly caused by pathogenic loss-of-function variants in SCN1A, in which case sodium channel-blocking ASMs, such as carbamazepine, should be avoided [1]. Gain-of-function SCN1A variants have also been associated with a more severe, neonatal-onset phenotype. First-line therapies include valproate, clobazam, stiripentol, fenfluramine, and cannabidiol, reflecting a move toward syndrome-specific pharmacologic strategies [1]. Despite treatment advances, DS remains associated with increased mortality, including risk of sudden unexpected death in epilepsy (SUDEP). Besides seizures, DS is accompanied by significant comorbidities including cognitive, motor, behavioral, and sleep impairments [18]. However, current ASMs do not improve these comorbidities, driving interest in emerging disease-modifying approaches such as antisense oligonucleotides (ASOs) for SCN1A DS [18].

Infantile Epileptic Spasms Syndrome (IESS)

IESS, formerly West syndrome, is a DEE historically characterized by a triad of spasms, hypsarrhythmia, and developmental regression. However, the latest ILAE classification recognizes atypical variants important for accurate diagnosis: spasms without hypsarrhythmia, hypsarrhythmia without spasms, and single-spasm variants lacking clustering [19]. IESS has over 200 etiologies spanning structural (such as hypoxic-ischemic encephalopathy), genetic (e.g., ARX, CDKL5, TSC1/TSC2), and metabolic categories, though approximately one-third of cases remain of unknown etiology [19]. Adrenocorticotropic hormone (ACTH) is the preferred treatment for non-TSC cases given the role of endogenous CRH as a proconvulsant, while vigabatrin is effective for TSC-associated IESS. Nevertheless, ACTH’s efficacy is incomplete, achieving spasm cessation in 55-75% of cases long-term. Regardless of treatment, prompt treatment initiation is critical to achieve better neurodevelopmental outcomes [19]. A summary of the key clinical features, etiologies, EEG findings, and treatment approaches of major atypical pediatric epilepsy syndromes is provided in Table 3.

**Table 3:** Summary of Key Atypical Pediatric Epilepsy Syndromes Data were synthesized from relevant ILAE classification position papers and syndrome-specific clinical reviews: EMAtS [3,13], LGS [3,14,15], FIRES [3,16], D/EE-SWAS [3,17], DS [1,2,18], and IESS [2,19]. ACTH: adrenocorticotropic hormone; ASMs: antiseizure medications; D/EE-SWAS: developmental and/or epileptic encephalopathy with spike-and-wave activation in sleep; DS: Dravet syndrome; EEG: electroencephalogram; EMAtS: epilepsy with myoclonic-atonic seizures; FIRES: febrile infection-related epilepsy syndrome; IESS: infantile epileptic spasms syndrome; LGS: Lennox-Gastaut syndrome; LOF: loss-of-function; TSC: tuberous sclerosis complex

Syndrome	Age of Onset	Etiology	Clinical Features	EEG Findings	Treatment Approaches
EMAtS	2-5 years	Genetic	Myoclonic-atonic seizures, developmental regression during exacerbations, absence and generalized tonic-clonic seizures	Generalized spike-wave discharges	Valproate
LGS	3-5 years	Structural, genetic, metabolic, or combined	Multiple seizure types, cognitive impairment	Generalized slow spike-and-wave complexes (≤2.5 Hz), paroxysmal fast activity during sleep	Multimodal therapy, including ASMs, dietary therapy, and surgical or neuromodulatory interventions
FIRES	School-aged	Usually cryptogenic; autoimmune or genetic etiologies may also be identified	Refractory status epilepticus after febrile illness	Multifocal discharges, beta-delta complexes	Immunotherapy, ketogenic diet, ASMs
D/EE-SWAS	Infancy, early childhood	Genetic (e.g. GRIN2A), structural	Cognitive, behavioral, and motor regression, sleep-activated seizures	Sleep-potentiated spike-wave activation	Corticosteroids, benzodiazepines, and other immunomodulatory therapies
DS	Infancy (<1 year)	SCN1A mutation (LOF)	Febrile seizures, developmental delay	Generalized spike-wave, photosensitivity	Valproate, clobazam, stiripentol, fenfluramine, cannabidiol
IESS	Infancy (<1 year)	Structural, genetic, metabolic	Spasms, hypsarrhythmia, developmental regression	Hypsarrhythmia may be present but is not required	ACTH for non-TSC cases; vigabatrin for TSC-associated IESS

Other atypical and diagnostically challenging presentations

Mechanisms of Immune-Mediated Epilepsies (IME)

Immune-mediated encephalitis has become a major cause of late childhood and adolescent epilepsy [1]. One mechanism consists of inflammatory cytokines increasing excitability and decreasing inhibition to decrease seizure threshold, such as in the case of FIRES. B-cell and autoantibody-mediated processes can also play a role by binding directly to receptors, yielding hyperexcitability and dysfunction, as seen in anti-NMDA receptor encephalitis (NMDARE). Lastly, T-cell-mediated inflammatory mechanisms can also perpetuate refractory seizures through neuronophagia and gliosis, as exemplified by Rasmussen syndrome (RS) [20].

RS and Antibody-Mediated Epilepsies

RS is an acquired progressive encephalitis of one hemisphere presenting with seizures, hemiparesis, and cognitive decline progressing to a chronic phase with permanent neurological damage [20]. Although the use of immunotherapy slows the disease, no specific antibody has been isolated, and hemispherectomy is considered the definitive cure [1].

NMDARE is the most common autoimmune encephalitis in children and one of the few autoimmune encephalitides that is often paraneoplastic in older children [20]. Diagnosis is made with the presence of at least two neuropsychiatric symptoms and CSF NMDAR antibody positivity. NMDARE is responsive to immunotherapy, with 70-80% of patients experiencing good outcomes at two years [20]. Anti-GABA-A receptor encephalitis is another important condition given its high seizure incidence, mostly generalized and often with SE. Additionally, anti-GAD65-associated conditions are T-cell mediated and unique in targeting intracellular antigens, in contrast to cell-surface antibodies, and are not being directly pathogenic [20].

Reflex Seizures in Children

Reflex seizures are epileptic events elicited by a specific afferent stimulus, ranging from simple extrinsic triggers (e.g., flickering lights, hot water) to complex intrinsic activities (e.g., reading, praxis) [21]. The ILAE formally recognizes four reflex epilepsy syndromes - POLE, other visual-sensitive epilepsies, primary reading epilepsy, and startle epilepsy - though reflex seizures also occur as traits within broader syndromes such as juvenile myoclonic epilepsy and EEM [3,21]. The genetic basis of reflex seizures is predominantly polygenic, though single-gene variants in CACNA1A, GNAO1, and NOVA2 have been identified in children with reflex seizures as predominant epileptic events [22]. Treatment of reflex seizures combines stimulus avoidance and lifestyle modifications (e.g., polarized glasses, screen distance adjustments) with broad-spectrum ASMs [21].

Sleep-Related Epilepsies in Children

The relationship between sleep and epilepsy is bidirectional: seizures and interictal epileptiform discharges (IEDs) during sleep disrupt sleep architecture, while poor sleep lowers seizure threshold [23]. Several childhood epilepsy syndromes are strongly sleep-related. SeLECTS, the most common focal epilepsy in children (6-7% of childhood epilepsies), presents with nocturnal focal seizures involving unilateral facial twitching, drooling, and dysarthria [23]. Sleep-related hypermotor epilepsy (SHE), formerly nocturnal frontal lobe epilepsy (NFLE), poses a diagnostic challenge due to semiological overlap with NREM parasomnias [23]. SHE’s distinguishing features include seizure stereotypy, abrupt onset/offset, and multiple episodes per night at variable times [24]. Genetically, SHE is associated with pathogenic variants in nicotinic acetylcholine receptor subunit genes (CHRNA4 and CHRNB2) and mTOR signaling gene DEPDC5, among others [23].

Diagnostic challenges

Overview of Diagnostic Challenges

Epilepsy misdiagnosis occurs in approximately 20% of cases, and this rate is particularly concerning in children, where age-dependent semiology and limited self-reporting complicate clinical assessment [25]. Diagnostic delay affects up to 77% of people with epilepsy because seizure signs go unrecognized by caregivers or clinicians. Epilepsy remains a clinical diagnosis following the ILAE stepwise framework: determining whether the event is epileptic, then classifying seizure type, epilepsy type, and syndrome. No single test is diagnostic in isolation. EEG epileptiform discharges without a supportive clinical history do not meet diagnostic criteria [25].

Nosological Overlap and EEG Limitations

Recognition of childhood syndromes requires careful analysis of seizure semiology, evolution, and the child's development, EEG, MRI, and genetic studies [3]. DEEs can evolve from one syndrome to another; for example, IESS can evolve to LGS or SeLECTS. In other syndromes, children with normal development can present with a severe encephalopathy followed by DRE, as seen in FIRES and HHE. Additionally, for some SeLFEs, there may be overlap with the idiopathic generalized epilepsies (IGEs) or even evolution to them [3]. All these factors can make accurate syndromic classification and diagnosis difficult. Furthermore, EEG sensitivity is imperfect in atypical presentations: a routine 30-minute recording captures epileptiform discharges in only 11% of patients after a first unprovoked seizure, compared with 72% for 24-hour ambulatory EEG [25]. Specific patterns can also mislead - centrotemporal spikes have 100% sensitivity but only 70% specificity to diagnose childhood epilepsy, with a positive predictive value of 58% [26]. These limitations underscore that no single EEG pattern is pathognomonic for a specific atypical syndrome and that findings must always be interpreted within the full clinical context.

Distinguishing Epileptic Seizures From Non-epileptic Mimics

Distinguishing epileptic seizures from non-epileptic paroxysmal events remains a persistent diagnostic challenge because of substantial overlap in clinical semiology. A 2026 systematic review and meta-analysis of 40 studies involving 3,123 children identified several semiological features that may help differentiate these events: tremor, asynchronous motor events, movements involving agonist-antagonist muscle groups, and akinetic semiology were predominantly associated with non-epileptic events, whereas tonic seizures and spasms were more frequently observed in epilepsy [27]. Although history and clinical examination form the fundamental basis of diagnosis, video-EEG remains the criterion standard for definitive diagnosis, while clinical semiology may help guide diagnostic prioritization before video-EEG is available [27]. To bridge this gap, smartphone video capture of events has emerged as a valuable and accessible diagnostic adjunct, and structured interview questionnaires are being developed to further improve discrimination at the point of first clinical contact [25].

Genetic Heterogeneity and Barriers to Timely Genetic Diagnosis

The genetic architecture of atypical pediatric epilepsies is remarkably heterogeneous. This heterogeneity means that identical phenotypes can arise from pathogenic variants in numerous different genes - for example, KCNT1 variants span multiple syndromes including early infantile DEE, focal epilepsy, IESS, and EIMFS [28]. The international Gene-STEPS study confirmed a 43% diagnostic yield with rapid trio genome sequencing in infantile epilepsy, with a median time from seizure onset to result of just 37 days [29]. However, genome sequencing remains unavailable in most clinical settings, and standard testing often takes months to years, during which infants may suffer progressive neurological sequelae from uncontrolled seizures [29]. These findings support current recommendations from the American Epilepsy Society endorsing a genome-wide approach as first-line genetic testing in early-onset epilepsies [29].

Emerging therapeutics

Novel ASMs

The therapeutic landscape for atypical pediatric epilepsies is rapidly expanding with newer ASMs being developed alongside advances in surgical, neurostimulation, and emerging genetic therapies. Recent advances include the approval and clinical development of cannabidiol for LGS, DS, and tuberous sclerosis complex (TSC); fenfluramine for LGS and DS; everolimus for TSC; and ganaxolone for cyclin-dependent kinase-like deficiency disorder [30]. Despite demonstrated reductions in seizure frequency with these therapies, seizure freedom has rarely been achieved in randomized controlled trials, highlighting the continued need for additional treatment options [30]. At the same time, emerging therapies are increasingly extending beyond conventional ASMs to include responsive and less-invasive neuromodulation approaches, genetic and related therapies, and focused ultrasound [30]. Investigational therapies highlighted at the Eighteenth Eilat Conference include bexicaserin, elsunersen, ETX101, radiprodil, RAP-219, relutrigine, vormatrigine, and zorevunersen, representing approaches directed at serotonergic signaling, glutamatergic pathways, ion channels, and genetic mechanisms [31]. Collectively, these agents illustrate a transition toward modulation of defined ion channel, serotonergic, and glutamatergic pathways rather than nonspecific neuronal inhibition.

Precision Therapies for Genetic DEEs

Precision therapies for DEEs are increasingly stratified by underlying molecular mechanisms, enabling targeted interventions across channelopathies, synaptic disorders, and metabolic epilepsies. Samanta et al. (2025) comprehensively reviewed current and emerging precision therapies for DEEs, organized by pathophysiological mechanisms [32]. Targeted approaches for channelopathies include ASOs and gene therapies for SCN1A-related DS (zorevunersen/STK-001, ETX101) as well as novel small molecules for other ion channel disorders. Neurotransmitter receptor dysfunction modulators include gaboxadol, radiprodil, and L-serine for GABA and glutamate receptor variants [32]. Synaptic dysfunctions are targeted via chemical chaperones for STXBP1-related disorders and Ras-Raf-MEK-ERK inhibitors for SYNGAP1 pathologies. The review also discusses mTOR inhibitors (everolimus) for TSC and ketogenic diet for GLUT-1 deficiency [32].

Gene-Targeted Therapies

ASOs are emerging as disease-modifying precision therapies for monogenic DEEs, modulating gene expression at the mRNA level through either RNase H1-mediated transcript degradation or splice-switching to upregulate functional protein from a haploinsufficient allele [33]. The most clinically advanced program targets DS, where zorevunersen - a splice-switching ASO that increases productive SCN1A expression - has shown reductions in convulsive-seizure frequency and sustained improvements in adaptive behavior over 36 months in 81 pediatric patients [34]. ASOs are being used beyond DS for other channelopathies, including SCN2A gain-of-function DEE and KCNT1-related disorders [32]. Nakayama et al. (2026) reported that intrathecal delivery of a KCNT1-targeting gapmer ASO in two infants with EIMFS led to significant seizure reduction, though both patients developed ventricular enlargement, highlighting a potential monitorable toxicity of intrathecal ASO delivery [35]. Regarding the broader applicability of these therapies, Sherrill et al. (2026) assessed 160 infants with genetic epilepsies and found that approximately 16% harbored variants currently amenable to ASO strategies [36].

Gene therapy approaches for pediatric epilepsy include viral vector-based strategies. ETX101, an AAV9-based gene therapy designed to increase NaV1.1 channel density in GABAergic interneurons, is in development for SCN1A-positive DS [37]. Emerging approaches include CRISPR-mediated transcriptional activation. Balestrini and Scheffer (2026) reviewed the full spectrum of newly approved and investigational drugs, RNA-based, and gene-based therapies for DS, noting that these advances signal a paradigm shift from symptomatic treatment to precision medicine [37].

Surgical and Neuromodulation Therapies

Epilepsy surgery remains the most effective intervention for drug-resistant pediatric epilepsy when a resectable epileptogenic zone can be identified. For instance, resective surgery has been effective for various LGS seizure types, with atonic seizures responding remarkably well to corpus callosotomies [38]. Ablation through laser interstitial thermal therapy has emerged as a minimally invasive alternative with significantly lower complication rates and shorter hospitalization than open surgery [39]. Resection or ablation are not options in the presence of multifocal or diffuse epilepsy, or when the epileptogenic zone overlaps with eloquent cortex. Instead, these patients could be candidates for neuromodulation including vagus nerve stimulation (VNS), deep brain stimulation (DBS), and responsive neurostimulation (RNS) [39].

Dietary Therapies

KDTs - including the classic ketogenic diet (cKD), modified Atkins diet (MAD), and low glycaemic index treatment (LGIT) - are established non-pharmacological interventions for drug-resistant pediatric epilepsies. A network meta-analysis of 12 RCTs (907 patients) found that all three dietary interventions were more efficacious than usual care for achieving at least a 50% reduction in seizure frequency, although ketogenic diet and MAD were associated with higher rates of adverse-event-related treatment discontinuation than usual care [40]. In children with DEEs specifically, add-on KD achieved significantly higher seizure response at six months than ASM adjustment alone (50.8% vs. 29.3%, p=0.001), with additional benefits in EEG improvement and developmental progress [41]. KDTs remain first-line for GLUT1 deficiency syndrome, with a reported 96% responder rate for SLC2A1 mutations compared with notably poor responses for CDKL5 (0% seizure freedom) and KCNT1 (2% seizure freedom), underscoring the importance of genotype-informed dietary therapy selection [42].

Discussion

Atypical pediatric epilepsies encompass a broad and evolving spectrum of disorders that challenge traditional diagnostic and therapeutic paradigms. Despite advances in ILAE classification and increasing recognition of genotype-phenotype correlations, a substantial proportion of children remain without a clearly defined epilepsy syndrome. Rather than reflecting a single diagnostic limitation, this uncertainty highlights the need to determine which newer diagnostic approaches are sufficiently validated and accessible for routine clinical practice.

The etiological diversity of atypical epilepsies - spanning genetic, structural, metabolic, and immune-mediated mechanisms - needs a multidisciplinary and increasingly personalized diagnostic approach. Early integration of prolonged EEG monitoring, high-resolution neuroimaging, and genome-wide sequencing has improved diagnostic yield, yet barriers to access continue to delay diagnosis and treatment in many settings. An important unresolved question is how these advances can be incorporated into routine care without widening existing disparities in access, particularly in resource-limited settings.

Therapeutically, the field is undergoing a paradigm shift from symptomatic seizure suppression toward mechanism-based and disease-modifying strategies. The development of precision therapies, including ASOs and gene-based interventions, represents a promising frontier, particularly for monogenic DEEs. However, many of these approaches remain investigational, and the available evidence varies substantially in maturity. Early-phase studies of therapies such as zorevunersen and KCNT1-targeted ASOs are encouraging, but larger controlled studies and longer follow-up are needed to establish efficacy, durability, and safety. Cost, scalability, and equitable access will also influence whether these therapies can be translated beyond highly specialized centers.

Ultimately, improving outcomes in atypical pediatric epilepsy will depend on earlier recognition, refined classification systems, and broader access to advanced diagnostic and therapeutic modalities. Future research should prioritize prospective studies that clarify which diagnostic tools provide meaningful changes in clinical management, establish the long-term safety and effectiveness of precision therapies, improve inclusion of patients with unclassified or genetically heterogeneous epilepsies in clinical trials, and address implementation barriers in resource-limited settings.

Limitations

As a narrative review, this study is subject to selection bias, as studies were chosen based on relevance rather than a systematic protocol. Although efforts were made to include high-quality and recent literature, some relevant studies may have been omitted. Additionally, the rapidly evolving nature of pediatric epilepsy, such as in the fields of genomics and precision therapeutics, means that some emerging data may not yet be fully captured. Variability in study design, population characteristics, and diagnostic criteria across the included studies also limits direct comparison of findings. Finally, much of the available literature originates from specialized centers in high-resource settings, which may limit generalizability to broader or resource-limited settings. Future iterations of this work would benefit from formal review by pediatric epilepsy specialists to further enhance clinical accuracy and applicability.

## Conclusions

Atypical pediatric epilepsies encompass a large group of disorders characterized by diverse etiologies, variable clinical presentations, and frequent resistance to standard ASMs. Advances in classification, genomic diagnosis, neuroimaging, and emerging targeted therapies have improved the ability to characterize and manage these disorders, but important gaps remain in diagnostic access, treatment evidence, and equitable implementation. Continued research is needed to determine which emerging diagnostic and precision-treatment approaches can be incorporated safely and effectively into routine pediatric epilepsy care.
